# The Facile Synthesis of Hollow CuS Microspheres Assembled from Nanosheets for Li-Ion Storage and Photocatalytic Applications

**DOI:** 10.3390/nano13091505

**Published:** 2023-04-28

**Authors:** Yiyang Zhao, Yonghui Shao, Hao Chen, Xinwen Luo, Xiaodi Liu

**Affiliations:** College of Chemistry and Pharmaceutical Engineering, Nanyang Normal University, Nanyang 473061, China

**Keywords:** CuS, hollow microsphere, nanosheet, lithium-ion batteries, photocatalyst

## Abstract

Herein, well-defined hollow CuS microspheres assembled from nanosheets were successfully synthesized through a facile solvothermal method. Hollow CuS microspheres have an average diameter of 1.5 μm; moreover, the primary CuS nanosheets have an ultrathin thickness of about 10 nm and are bound by {0001} polar facets. When used as anodes for lithium-ion batteries (LIBs), hollow CuS microspheres exhibit excellent electrochemical properties, including a large discharge capacity (610.1 mAh g^−1^ at 0.5 C), an excellent rate capability (207.6 and 143.4 mAh g^−1^ at 1 and 5 C), and a superior cyclic stability (196.3 mAh g^−1^ at 1 C after 500 cycles). When used as photocatalysts for Rhodamine B (RhB), hollow CuS microspheres can degrade more than 99% of the initial RhB within 21 min. These excellent Li-ion storage properties and photocatalytical performances are attributed to their unique hierarchical hollow structure.

## 1. Introduction

Recently, with energy and environment issues becoming more and more prominent, developing high-performance electrochemical energy storage (EES) devices and high-efficiency pollution control technologies has attracted tremendous research attention [1,2]. For the former, lithium-ion batteries (LIBs) are extensively used as critical EES devices for portable electronic devices and electrified vehicles, and the exploration of effective anode materials may result in high-performance LIBs [3,4]. For the latter, photocatalytic treatment is a promising method for eliminating the organic and toxic pollutants in water, and photocatalysts are a key factor that affects the photocatalytic activity [5,6].

Until now, several advanced materials, such as CuS, Fe_2_O_3_, TiO_2_, Nb_2_O_5_, and so on, have not only served as anodes for LIBs, but have also acted as photocatalysts for wastewater treatment [5,7,8,9,10,11]. Among them, CuS has received increasing interest due to its unique optical and electrical properties. When used as an anode for LIBs, CuS possesses exceptional qualities, including a high theoretical capacity (561 mAh g^−1^), a good metal-like electronic conductivity (1 × 10^−3^ S cm^−1^), a special layered structure with large interlayer spacing, large abundant resources, and high safety [12,13]. However, as a kind of typical 2D material, CuS has a strong tendency to stack with each other [14]. Thus, the length of the charge transport paths would be increased and the electrochemically active area would be decreased, leading to a limited rate capability and cycle stability.

On the other hand, as a p-type semiconductor with a favorable bandgap (ca. 2.2 eV), CuS has received considerable attention for the treatment of toxic organic dyes [15,16]. Its photocatalytic property is closely related to the photogenerated electron–hole pairs of the photocatalyst, and so much effort has been focused on improving its photocatalytic performance by increasing the surface area, light absorptivity, and collection of charge carriers [8]. In this regard, the design of CuS for excellent Li-ion storage performance and photocatalytic activity is expected.

The construction of CuS nanomaterials is an efficient method for improving their physico-chemical properties, owing to their small size and large specific surface area [17,18]. Nevertheless, nanomaterials have a strong tendency to aggregate with each other due to their large surface energy, thus leading to poor performance. The construction of hierarchical micro/nanostructures assembled from nanoscale building blocks is an effective approach to prohibiting the agglomeration of nanomaterials; moreover, hierarchical structures possess special physical and chemical properties [18,19,20,21]. Among a wide range of hierarchical superstructures, hollow microspheres composed of nanosheets can supply a high surface area, low material density, good surface penetration, high charge transport rate, and good light absorptivity [8,22,23,24]. Thus, a hierarchical hollow microsphere is an ideal structure for obtaining excellent electrical and optical properties. Until now, several methods, including the templates sacrificial technique [25], Kirkendall effect [26], and precursor transformation [27], have been employed to prepare these hierarchical hollow microspheres. However, it still remains a challenge to explore facile and effective approaches to constructing well-defined hollow CuS microspheres.

Herein, nanosheet-assembled hollow CuS microspheres were prepared by a facile solvothermal method without using templates. By simply changing the volume ratio of deionized water to glycerol, uniform hollow microspheres can be obtained. Their hollow structure is beneficial for the penetration of electrolyte into electrodes and supplies extra active sites for the storage of Li^+^ ions. Moreover, the special hollow structure significantly enhances the light absorption capabilities. Accordingly, hollow CuS microspheres possess excellent Li-ion storage properties and photocatalytic activity.

## 2. Materials and Methods

### 2.1. Synthesis of Hollow CuS Microspheres

All the chemicals were purchased from Shanghai Aladdin Bio-Chem Technology Co., Ltd. (Shanghai, China). In the typical synthesis, 5 mmol CuSO_4_·5H_2_O and 10 mmol CH_3_CSNH_2_ (TAA) were added into 15 mL of a deionized water–glycerol solvent (with a volume ratio of deionized water to glycerol, R_w/g_, of 5:1). After being stirred for 15 min, the solution was moved into a Teflon-lined autoclave (30 mL) and maintained at 150 °C for 9 h. After the samples were centrifuged, washed, and dried, hollow CuS microspheres (denoted by S-1) were obtained. In addition, to inspect the influence of the solvent on the morphology of the products, the same procedure as that for the synthesis of S-1 was used for the fabrication of S-2 and S-3, except that the R_w/g_ values were 1:0 and 2:1, respectively.

### 2.2. Materials Characterization

The crystal structures of the products were analyzed using a Rigaku D/max 2500V/PC X-ray diffractometer (XRD, Rigaku Corporation, Tokyo, Japan) using Cu Kα as rediation. The morphologies and nanostructures of the samples were researched using SU8010 field-emission scanning electron microscopy (FESEM, Hitachi, Ltd., Tokyo, Japan) and JEM-2100F transmission electron microscopy (TEM, JEOL Ltd., Tokyo, Japan). The Brunauer–Emmett–Teller (BET) special surface area of the sample was researched using the N_2_ adsorption–desorption isotherms on a Micromeritics Autosorb-iQ apparatus (Quantachrome Inc., Florida, FL, USA). The X-ray photoelectron spectra (XPS, Thermo Fisher Scientific Inc., Massachusetts, MA, USA) were researched using a Thermo ESCALAB 250XI electron spectrometer.

### 2.3. Electrochemical Measurement

S-1, acetylene black, and polyvinylidene fluoride with a ratio of 70:20:10 wt% were mixed into N-methyl-2-pyrrolidone. The slurry was uniformly coated on Cu foils and then dried in vacuum at 120 °C for 12 h. Subsequently, the electrodes were assembled into CR2025 coin-type cells in an Argon-filled glove box. Li was served as the counter and reference electrode. The electrolyte was 1 mol·L^−1^ LiPF_6_ in ethylene carbonate, ethyl methyl carbonate, and diethyl carbonate (1:1:1 vol%). Cyclic voltammetry (CV) measurements were carried out on a CHI660D Electrochemical Workstation (Shanghai Chenhua Instrument Ltd., Shanghai, China).

### 2.4. Photocatalytic Test

The photocatalytic activities of S-1 and S-2 were researched using the photodegradation of Rhodamine B (RhB), using a 500 W Xe lamp equipped with a UV cut-off filter (λ > 420 nm) as the irradiation source. In the typical photocatalytic experiment, 10 mg of the photocatalyst was added into 100 mL of a RhB dye solution (10 mg/L). Prior to irradiation, the obtained suspension was magnetically stirred for 30 min in the dark to reach an adsorption–desorption equilibrium. In the following photocatalytic reaction, 4 mL of the suspension was collected at a given time interval and centrifuged (10,000 rpm, 5 min) to remove the photocatalyst. The contents of RhB were tested by detecting the values of feature absorbance of 553 nm on a Lambda 650 s UV-Vis spectrophotometer (Perkin Elmer Instruments Inc., Massachusetts, MA, USA).

## 3. Results

### 3.1. Structure Characterization

**Figure 1:**
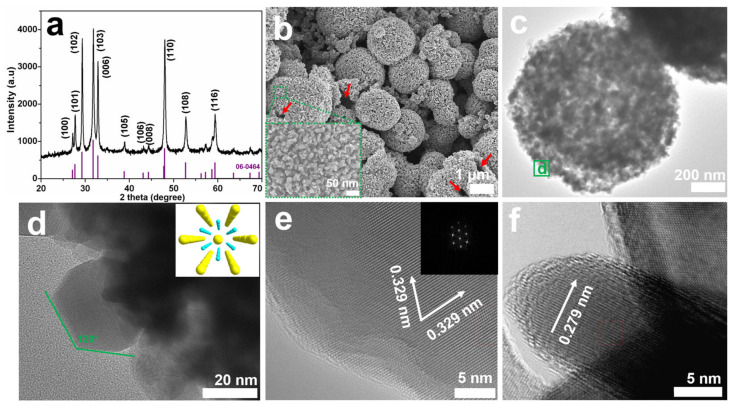
(**a**) XRD pattern of S-1, and the purple lines are the standard XRD reflections of hexagonal CuS; (**b**) FESEM image and (**c**) TEM image of S-1; (**d**) high-magnification TEM image of S-1 originating from the green square in (**c**), the inset is the atomic arrangement of hexagonal CuS viewed along the *c*-axis (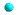 Cu, 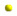 S); (**e**) HRTEM image of a primary nanosheet, the inset is the corresponding FFT pattern from the [001] direction; and (**f**) HRTEM image of a nanosheet standing perpendicular to the substrate.

The composition, morphology, and nanostructure of S-1 were researched using XRD, SEM, TEM, and HRTEM. Figure 1a displays the XRD pattern of S-1. Obviously, the positions and intensities of all the diffraction peaks are in good agreement with the hexagonal CuS phase (JCPDS No. 06-0464). The strong peaks with 2θ values of 29.3°, 31.8°, 32.9°, and 47.9° correspond to the (102), (103), (006), and (110) facets of the hexagonal CuS, respectively. No other peaks for impurities such as Cu_2_S or CuO are observed, indicating that S-1 is highly pure. In addition, the sharp diffraction peaks reveal the good crystalline nature of S-1. From the FESEM image (Figure 1b), it can be observed that the products are well-defined microspheres with an average diameter of about 1.5 μm. As shown in the high-magnification FESEM image (inset of Figure 1b), the microspheres are composed of small hexagonal nanosheets with an edge length of about 30 nm and a thickness of about 10 nm. As indicated by the red arrows in Figure 1b, the microspheres possess a hollow structure.

In the corresponding TEM image (Figure 1c), the contrast between the dark edge and light center further affirms the hollow interior void of S-1 [22]; furthermore, the high-magnification TEM image (Figure 1d, taken from the green square d in Figure 1c) clearly points out that these primary nanosheets have symmetrical hexagonal shapes. The HRTEM image taken of the typical hexagonal nanosheet (Figure 1e) exhibits continuous lattice fringes, further showing the good crystallization of S-1. Two clear lattice fringes have similar lattice fringes of ca. 0.329 nm, corresponding to the *d*-spacing of the (100) and (010) facets. Additionally, it should be mentioned that the hexagonal symmetry spots in the corresponding fast Fourier transform (FFT) pattern (inset of Figure 1e) are consistent with the atomic arrangement of the hexagonal CuS along the c-axis (inset of Figure 1d). Furthermore, the (006) plane with a lattice spacing of ca. 0.279 nm can be clearly seen from the HRTEM image of a nanosheet standing on the substrate (Figure 1f). Therefore, it can be deduced that these sheet-like building blocks are mainly bound by {0001} polar facets [28]. Owing to its hierarchical hollow microstructure, S-1 has a large specific surface area of 50.8 m^2^ g^−1^ (Figure S1), which is beneficial for improving Li-ion storage properties and photocatalytic performance.

**Figure 2:**
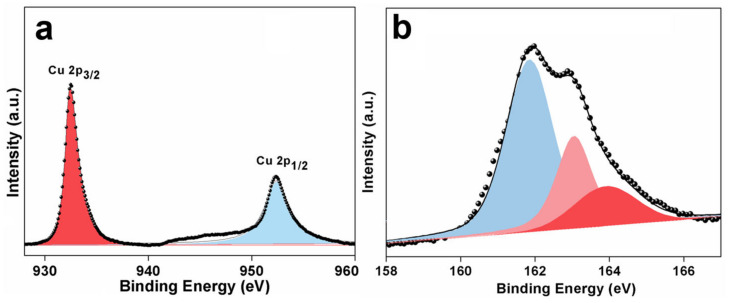
(**a**) Cu 2p, and (**b**) S 2p XPS spectra of S-1.

XPS was further used to inspect the surface composition and chemical state of S-1. The signals, including the Cu, S, C, and O elements, can be detected from the wide-scan XPS survey of S-1 (Figure S2). The O1s signal at 532.1 eV is relatively weak, revealing the adsorption of oxygen on the surface of S-1 [29]. Moreover, the Auger line of Cu (Cu LMM) appears at 569.1 eV, corresponding to a kinetic energy of 917.5 eV, indicating that the Cu element in CuS is presented in the form of Cu^2+^ [30]. Figure 2a,b are the high-resolution Cu 2p and S 2p XPS spectra, respectively. In Figure 2a, the two main peaks at around 932.5 and 952.4 eV can be ascribed to the Cu 2p_3/2_ and Cu 2p_1/2_ of the Cu^2+^ in CuS, respectively, and these values are for the Cu^2+^ oxidation state in CuS [15,31]. Meanwhile, as shown in Figure 2b, the S 2p core-level spectrum of S-1 can be fitted to three peaks at 161.9, 162.8, and 163.9 eV. The first two peaks can be assigned to the Cu-S bond and the last one is attributed to the S-S bond [12]. According to the XPS results, the atomic ratio of Cu to S is about 47.7:52.3. The superfluous S may be caused by the excess amount of TAA.

### 3.2. Formation Mechanism

**Figure 3:**
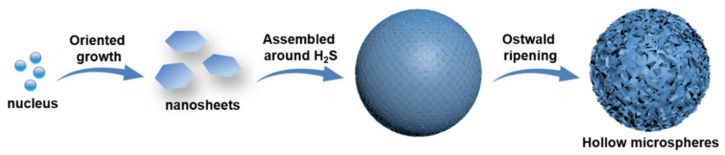
Illustration of the possible formation mechanism of S-1.

During the synthesis, the Cu^2+^ ions first react with TAA to form [Cu(TAA)_2_]^2+^ complex ions (Cu^2+^ + 2TAA → [Cu(TAA)_2_]^2+^) and then these [Cu(TAA)_2_]^2+^ ions are converted into CuS nuclei and H_2_S gas ([Cu(TAA)_2_]^2+^ + 4H_2_O → CuS↓ + 2H^+^ + 2CH_3_COONH_4_ + H_2_S↑). It is known that the growth orientations of crystals are closely related to their intrinsic crystal structures [32]. According to the XRD result (Figure 1a), the crystal structure of hexagonal CuS belongs to the P63/mmc space group, with lattice parameters of *a* = *b* = 3.793 Å and *c* = 16.346 Å. In the crystal structure of hexagonal CuS, two kinds of close-packed atom layers ([CuS_3_] and [Cu_3_S]) are alternatively arranged along the [001] direction. Specifically, in the [CuS_3_] layer, each Cu atom is coordinated by four S atoms to form a tetrahedron, whereas each Cu atom in the [Cu_3_S] layer is coordinated by three S atoms in a triangle [28]. Owing to this anisotropic characteristic, CuS nuclei prefer to grow in the *ab* planes rather than along the *c*-axis, resulting in the generation of hexagonal sheet-like building blocks [33]. During the following growth process, to minimize the surface energies, the primary CuS nanosheets tend to assemble around the interfaces of H_2_S gas bubbles [34,35]. Finally, a hierarchical hollow structure can be formed. The possible formation mechanism of S-1 is illustrated in Figure 3.

**Figure 4:**
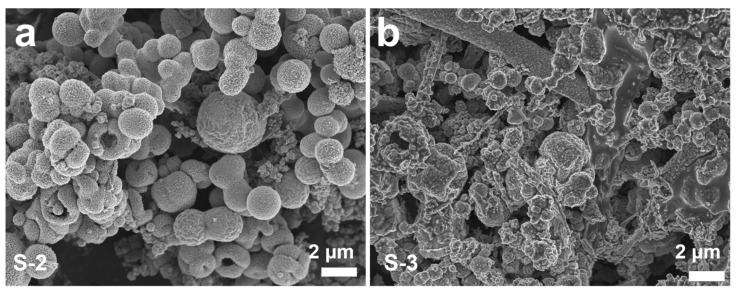
FESEM images of CuS prepared at different solvents with the other reaction parameters uncharged: (**a**) 15 mL deionized water (S-2); and (**b**) 10 mL deionized water and 5 mL glycerol (S-3).

In the synthetic system, it is deduced that glycerol content plays an important role in the formation of S-1. Glycerol has a considerably higher viscosity than water (243 vs. 3 mPa s), so the primary CuS nanosheets have sufficient time to search for the low-energy configuration interface and assemble into a well-defined hollow microstructure in the deionized water–glycerol solvent [36]. Controlled experiments with different solvent components are conducted to verify the influence of the solvent on the structures of the products. As shown in Figure S3, the products prepared with different solvents are CuS with high purity. As the solvent contains only 15 mL of deionized water, the products (S-2) are composed of hollow microspheres and irregular aggregates (Figure 4a). Owing to the low viscosity of water, the primary nanosheets can be quickly aggregated with each other. Finally, some nanosheets are aggregated into hollow microspheres with a thick shell, and the others are assembled into irregular aggregates. As the content of the glycerol is increased to 5 mL (S-3), the solvent has an increased viscosity, which is not only averse to the anisotropic growth of hexagonal building blocks, but also hinders the formation of uniform microstructures. Hence, the products are mainly CuS messy aggregates (Figure 4b). The detailed experimental conditions and corresponding morphologies are summarized in Table S1.

### 3.3. Li-Ion Storage Properties

**Figure 5:**
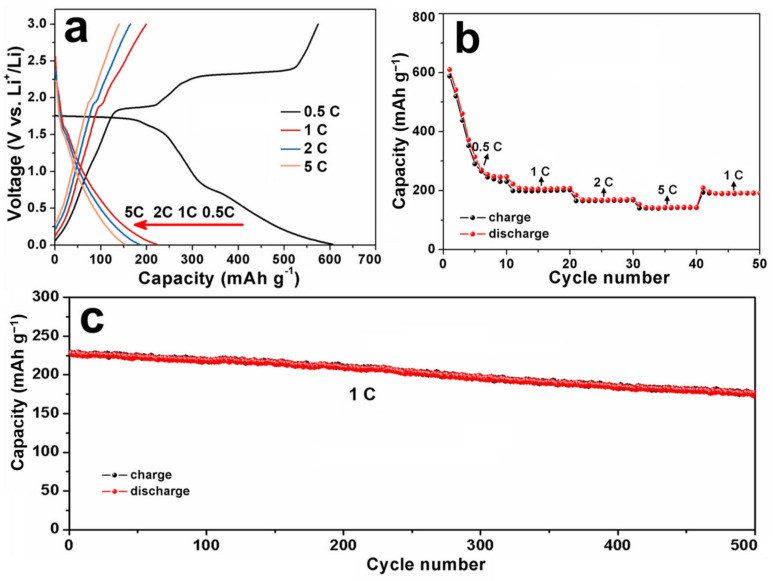
(**a**) The 1st discharge–charge curves of S-1 at different current densities; (**b**) rate capability; and (**c**) cycling performance of S-1.

The cyclic voltammogram (CV) curve of S-1 was tested at a scan rate of 0.1 mV s^−1^ over a voltage range of 0.01–3.0 V (vs. Li^+^/Li), and the result of this is shown in Figure S4. Similar to other papers [37,38], the reduction peak in the discharge process represents the conversion of Li*_x_*CuS (CuS + *x*Li^+^ + *x*e^−^ → Li*_x_*CuS) and the oxidation peaks in the charge process are associated with the formation of CuS. The Li-ion storage properties of S-1 were researched via the galvanostatic discharge–charge method. Figure 5a shows the first charge–discharge curves of S-1 in the voltage range of 0.01–3.0 V (vs. Li^+^/Li) at different current densities. As the current densities are 0.5, 1, 2, and 5 C, the first discharge capacities are 610.1, 230.7, 194.9, and 158.9 mAh g^−1^, respectively. The rate capability of S-1 was studied using a progressive discharge and charge from 0.5, 1 to 2, and 5 C for 10 cycles at each current density. As shown in Figure 5b, the anode exhibits excellent rate capacities of 230.6 to 207.6, 170.2, and 143.4 mAh g^−1^ at 0.5, 1, 2, and 5 C, respectively. Furthermore, the discharge capacity returns to 192.3 mAh g^−1^ as the current density is reduced to 1 C. These results indicate that the anode has an excellent rate performance and good structural stability at a high current density. The cyclic stability of S-1 was also investigated. The anode was first activated at a low current rate of 0.5 C for 10 cycles, and then the anode was repeatedly charged and discharged at 1 C. As shown in Figure 5c, S-1 exhibits a high reversible capacity of 196.3 mAh g^−1^ after 500 cycles, keeping a capacity retention of 88.2% compared to the first cycle. Additionally, compared to other copper-sulfide-based anodes [39,40,41,42,43,44], S-1 shows comparable Li-ion storage properties (Table S2).

Such results indicate that S-1 possesses excellent Li^+^ storage properties, which may be attributed to its unique structure. At first, its hierarchical hollow structure is beneficial for the penetration of electrolyte into electrode and it can supply extra active sites for the storage of Li^+^ ions. Thus, S-1 has a high discharge capacity [22,45]. Secondly, it is known that the time for the Li^+^/e^−^ diffusion in the electrode is proportional to the diffusion length. The primary CuS nanosheets have a thin thickness (ca. 10 nm), so the transfer rate of Li^+^ and e^−^ can be effectively enhanced, resulting in a good rate capacity [20,46,47]. Thirdly, the unique architecture of S-1 maintains the mechanical stability of the electrode during the discharge and charge processes, leading to an obvious improvement in its cycling stability.

### 3.4. Photocatalytic Performance

S-1 can be used as a photocatalyst for the degradation of organic dyes (e.g., RhB and MB) due to its unique hierarchical hollow structure. The photocatalytic performance of S-1 was tested by the photocatalytic degradation of RhB under visible light irradiation. As a comparison, S-2 was also tested under the same conditions. In the photocatalytic process [15,48], under the irradiation of visible light, CuS yields a pair of electrons (e^−^) and holes (h^+^) (CuS + h*υ* → e^−^ + h^+^). Then, the as-formed electrons leap into the bottom of the conduction band (CB) and react with O_2_ on the surface of the photocatalyst to form a superoxide radical (e^−^ + O_2_ → ·O_2_^−^). In addition, the holes stay at the top of the valence band (VB) and react with H_2_O to generate hydroxyl radicals (h^+^ + H_2_O → ·OH + H^+^). Finally, the active substances (O_2_^−^ and ·OH^−^) can oxidize RhB into smaller molecules (CO_2_, H_2_O, etc.).

**Figure 6:**
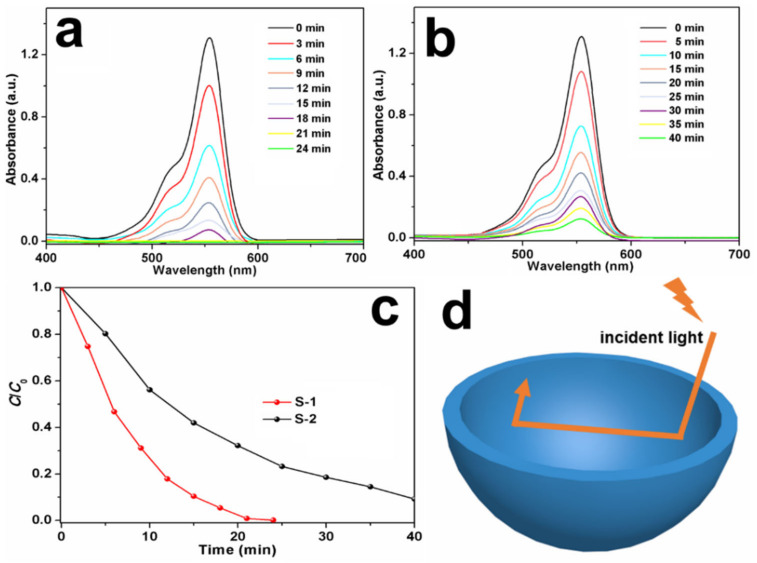
Time-dependent absorption spectra of RhB solutions in the presence of (**a**) S-1 and (**b**) S-2; (**c**) photodegradation rate of RhB solution by using S-1 and S-2 as photocatalysts; and (**d**) schematic illustration of the improved photocatalytic activity of S-1.

Figure 6a,b display the time-dependent absorption spectra of RhB aqueous solutions containing S-1 and S-2, respectively. With an increase in the irradiation time, the maximum absorption of RhB at 550 nm decreases, suggesting its decomposition. Figure 6c represents the photodegradation rates of the RhB aqueous solutions by using S-1 and S-2 as photocatalysts. Clearly, S-1 degrades more than 99% of the initial RhB within 21 min, whereas with S-2, only 68.3% of the RhB is decomposed after 20 min of irradiation, and 90.5% of the RhB can be decomposed after 40 min. Thus, S-1 exhibits stronger photocatalytic activity than S-2. Compared to some other metal sulfides [15,48,49,50,51,52,53], S-1 also exhibits an excellent photocatalytic performance (Table S3). Furthermore, it has been reported that copper sulfide photocatalysts suffer from photocorrosion under light irradiation, in which S^2−^ can be oxidized by h^+^ into S° or SO_4_^2−^, thus decreasing the photocatalytic activity [54,55]. Accordingly, the recycled photocatalytic test was carried out to research the stability of S-1 (Figure S5). It can be found that the degradation rate of S-1 slightly decreases by 2.1% after eight cycles, suggesting that S-1 has a good cycling stability.

It is deduced that the outstanding degradation efficiency of S-1 stems from the following reasons. Firstly, S-1 has a large specific surface area, so it conveniently absorbs more photons and produces plenty of electron–hole pairs [56]. Secondly, as shown in Figure 6d, the hollow microspheres can reflect the incident light between the inner voids and shells of S-1, enhancing its light absorption capabilities [57,58]. Thirdly, it is known that the e^−^/h^+^ recombination problem significantly affects the photocatalytic performance of photocatalysts. As for S-1, the ultrathin thickness of the primary nanosheets provides a short distance for the e^−^ transfer from the interior to the surface; moreover, the charge transfer is facilitated by the high crystallinity of the nanosheets. Hence, the h^+^/e^−^ recombination in the bulk is suppressed and the photocatalytic performance of S-1 is enhanced accordingly [54,59]. Fourthly, the exposed facet also plays an important part in the photocatalytic properties of S-1 [60,61]. According to the HRTEM results, the flat surfaces of the primary nanosheets are exposed by the {0001} facets, while the lateral surfaces are exposed by the {10-10} facets. The self-constructed {0001}/{10-10} facet junctions in the CuS nanosheets can accelerate the charge carriers’ separation and transfer [61]. Thus, the recombination of h^+^ and e^−^ is effectively prevented, endowing S-1 with a good photocatalytic activity and stability for organic pollutant degradation.

## 4. Conclusions

In summary, we report the solvothermal syntheses, formation mechanisms, Li-ion storage properties, and photocatalytic performances of hollow CuS microspheres. Owing to the synergistic effects of their hollow structures, ultrathin nanosheets, special exposed facets, and hierarchical superstructures, hollow CuS microspheres have improved electrochemical and photocatalytic properties. At 0.5 C, hollow CuS microspheres exhibit a high discharge capacity of 610.1 mAh g^−1^ and they can retain a high capacity of 143.4 mAh g^−1^ even at 5 C. In addition, after 500 cycles at 1 C, they exhibit a high discharge capacity of 196.3 mAh g^−1^. On the other hand, hollow CuS microspheres degrade more than 99% of RhB within 21 min. Hence, hollow CuS microspheres show potential applications as anodic material for LIBs and photocatalysts for the treatment of organic dyes.

## Data Availability

Data sharing is not applicable to this article.

## Supplementary Materials

The following supporting information can be downloaded at: https://www.mdpi.com/article/10.3390/nano13091505/s1, Figure S1: N_2_ adsorption-desorption isotherm of S-1; Figure S2: Wide-scan XPS survey of S-1; Figure S3: XRD patterns of S-2 and S-3; Figure S4: CV curve of S-1 at a scan rate of 0.1 mV s^−1^; Figure S5: The photocatalytic cycling stability of S-1 for the degradation of RhB; Table S1: The detailed experimental conditions and corresponding morphologies of samples; Table S2: The comparison of the electrochemical performance of S-1 and other previously reported copper sulfides-based anodes for LIBs; Table S3: The comparison of the photocatalytic performance of S-1 and other previously reported metal sulfides for the degradation of organic dyes.
