# Supplementary material for: The Facile Synthesis of Hollow CuS Microspheres Assembled from Nanosheets for Li-Ion Storage and Photocatalytic Applications
Source: Nanomaterials (Basel). 2023 Apr 28;13(9):1505. doi: 10.3390/nano13091505 (PMC10179783; doi:10.3390/nano13091505)
Supplement: Supplementary file 1 [file nanomaterials-13-01505-s001.zip › nanomaterials-2323161-supplementary.pdf]

nanomaterials

Supporting Information of

The Facile Synthesis of Hollow CuS Microspheres Assembled from Nanosheets for Li-Ion Storage and Photocatalytic Applications

Yiyang Zhao, Yonghui Shao, Hao Chen, Xinwen Luo and Xiaodi Liu *

College of Chemistry and Pharmaceutical Engineering, Nanyang Normal University, Nanyang 473061, China
* Correspondence: 20122029@nynu.edu.cn; Tel.: +86-377-63513540

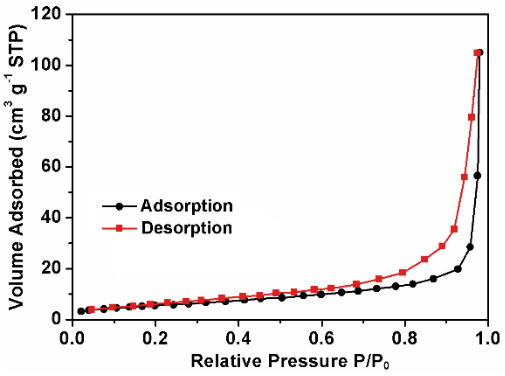

Figure S1. N_2 adsorption-desorption isotherm of S-1.

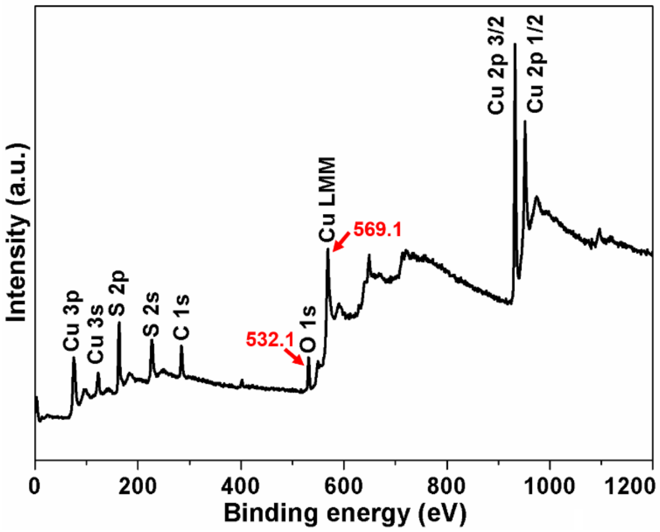

Figure S2. Wide-scan XPS survey of S-1.

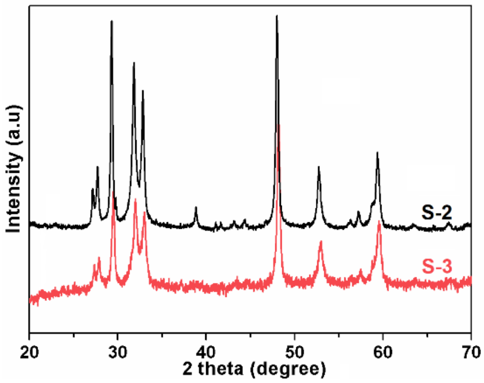

Figure S3. XRD patterns of S-2 and S-3.

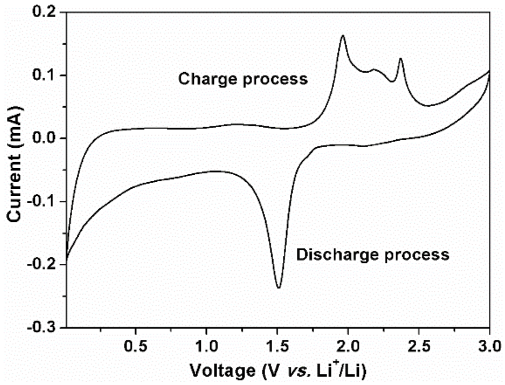

Figure S4. CV curve of S-1 at a scan rate of 0.1 mV s^{-1}.

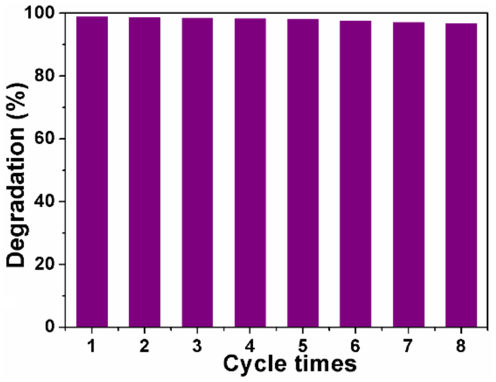

Figure S5. The photocatalytic cycling stability of S-1 for the degradation of RhB.

Table S1. The detailed experimental conditions and corresponding morphologies of samples.

Samples	Deionized water (mL)	Glycerol (mL)	Temperature (°C)	Time (h)	Morphology
**S-1**	12.5	2.5	150	9	Hollow microsphere
**S-2**	15.0	0.0	150	9	Hollow microsphere and irregular aggregates
**S-3**	10.0	5.0	150	9	Messy aggregates

Table S2. The comparison of the electrochemical performance of S-1 and other previously reported copper sulfides-based anodes for LIBs.

Samples	Discharge capacity	Cyclability	Ref.
S-1	610.1 mAh g^{-1} (0.5 C)	196.3 mAh g^{-1}/500th (1 C)	In this work
CuS microspheres	258 mAh g^{-1} (0.1 C)	~62 mAh g^{-1}/50th (0.1 C)	[1]
Tubular dandelion-like CuS superstructures	500 mAh g^{-1} (0.56 A g^{-1})	390 mAh g^{-1}/10th (0.56 A g^{-1})	[2]
Sphere-like CuS hierarchical structures	590 mAh g^{-1} (0.1 A g^{-1})	100 mAh g^{-1}/10th (0.1 A g^{-1})	[3]
Cu_xS/rGO nanocomposites	325 mAh g^{-1} (0.2 A g^{-1})	250 mAh g^{-1}/200th (0.2 A g^{-1})	[4]
Cu_2S nanoparticle	320 mAh g^{-1} (0.5 C)	125 mAh g^{-1}/50th (1 C)	[5]
Cu_2S/C composite	~285 mAh g^{-1} (0.5 C)	270 mAh g^{-1}/300th (0.2 C)	[6]

[1] B. Zhang, X.W. Gao, J.Z. Wang, S.L. Chou, K. Konstantinov, H.K. Liu, CuS nanoflakes, microspheres, microflowers, and nanowires: Synthesis and lithium storage properties, J. Nanosci. Nanotechnol., 2013, 13, 1309-1316.

[2] Z. Hosseinpour, A. Scarpellini, S. Najafishirtari, S. Marras, M. Colombo, A. Alemi, M.D. Volder, C. George, V. Lesnyak, Morphology-dependent electrochemical properties of CuS hierarchical superstructures, ChemPhysChem, 2015, 16, 3418-3424.

[3] Y. Han, Y. Wang, W. Gao, Y. Wang, L. Jiao, H. Yuan, S. Liu, Synthesis of novel CuS with hierarchical structures and its application in lithium-ion batteries, Powder Technol., 2011, 212, 64-68.

[4] Y. Zhang, K. Li, Y. Wang, J. Zeng, P. Ji, J. Zhao, Copper sulfide microspheres wrapped with reduced graphene oxide for high-capacity lithium-ion storage, Mater. Sci. Eng. B, 2016, 213, 57-62.

[5] Y. Fu, A. Manthiram, Electrochemical properties of Cu_2S with ether-based electrolyte in Li-ion batteries, Electrochim. Acta, 2013, 109, 716-719.

[6] F. Han, W.C. Li, D. Li, A.H. Lu, In situ electrochemical generation of mesostructured Cu_2S/C composite for enhanced lithium storage: Mechanism and material properties, ChemElectroChem, 2014, 1, 733-740.

Table S3. The comparison of the photocatalytic performance of S-1 and other previously reported metal sulfides for the degradation of organic dyes.

Samples	Dye	Dye concentration (mg/L)	Dye volume (mL)	Time (min)	Photodegradation (%)	Ref.
S-1 (0.1 g)	RhB	10	100	21	99	In this work
CuS spherical (0.1 g)	RhB	2.5	200	30	90	[1]
CdS decorated CuS microflower (0.3 g)	MO	10	50	30	91	[2]
CuS microflowers (0.05 g)	MB	5	30	25	98	[3]
CuS nanoflowers (0.1 g)	MB	20	100	90	39	[4]
SnS_2-RGO composite (0.05 g)	RhB	10	50	120	100	[5]
CdS/ZnS nanocomposites (0.2 g)	RhB	10	200	35	96.1	[6]
Flower-shaped CuS/Bi_2WO_6 nanosphere (0.1 g)	RhB	20	150	50	90	[7]

[1] Y. Zhang, B. Zhang, Z. Ge, L. Zhu, Y. Li. Preparation by solvothermal synthesis, growth mechanism, and photocatalytic performance of CuS nanopowders, Eur. J. Inorg. Chem., 2014, 14, 2368-2375.

[2] X. Deng, C. Wang, H. Yang, M. Shao, S. Zhang, X. Wang, M. Ding, J. Huang, X. Xu, One-pot hydrothermal synthesis of CdS decorated CuS microflower-like structures for enhanced photocatalytic properties, Sci. Rep., 2018, 7, 3877.

[3] K. Seo, K. Sinha, E. Novitskaya, O.A. Graeve, Polyvinylpyrrolidone (PVP) effects on iron oxide nanoparticle formation, Mater. Lett., 2018, 25, 203-206.

[4] H. Wu, Y. Li, Q. Li, Facile synthesis of CuS nanostructured flowers and their visible light photocatalytic properties, Appl. Phys. A, 2017, 123, 196.

[5] P. Chen, Y. Su, H. Liu, Y. Wang, Interconnected tin disulfide nanosheets grown on graphene for Li-ion storage and photocatalytic applications, ACS Appl. Mater. Interfaces, 2013, 5, 12073-12082.

[6] K. Zhang, Ling Jin, Y. Yang, K. Guo, F. Hu, Novel method of constructing CdS/ZnS heterojunction for high performance and stable photocatalytic activity, J. Photoch. Photobio. A, 2019, 380, 11859.

[7] H. Zhou, J. Guo, N. Fang, J. Liang, T. Shen, S. Yuan, Investigation of photocatalytic performance of CuS/Bi_2WO_6 and degradation pathway of RhB in water, J. Water Supply Res. T., 2020, 69, 145-159.
